# Lifelong Aerobic Exercise Alleviates Sarcopenia by Activating Autophagy and Inhibiting Protein Degradation via the AMPK/PGC-1α Signaling Pathway

**DOI:** 10.3390/metabo11050323

**Published:** 2021-05-18

**Authors:** Jiling Liang, Hu Zhang, Zhengzhong Zeng, Liangwen Wu, Ying Zhang, Yanju Guo, Jun Lv, Cenyi Wang, Jingjing Fan, Ning Chen

**Affiliations:** 1 Graduate School, Wuhan Sports University, Wuhan 430079, China; ljl19930210@163.com (J.L.); 17858503393@163.com (H.Z.); xxbbgdh@163.com (Z.Z.); guoyj_2520@163.com (Y.G.); lj864145056@163.com (J.L.); 2 Tianjiu Research and Development Center for Exercise Nutrition and Foods, Hubei Key Laboratory of Exercise Training and Monitoring, College of Health Science, Wuhan Sports University, Wuhan 430079, China; hancock120@163.com; 3 School of Physical Education, Hanshan Normal University, Chaozhou 521041, China; xibeiyu@126.com; 4 School of Physical Education and Sports Science, Soochow University, Suzhou 215021, China; cenyiwang@126.com

**Keywords:** lifelong aerobic exercise, sarcopenia, autophagy, apoptosis, mitochondrial quality control, ubiquitin-proteasome system

## Abstract

Sarcopenia is an aging-induced syndrome characterized by a progressive reduction of skeletal muscle mass and strength. Increasing evidence has attested that appropriate and scientific exercise could induce autophagy or optimize the functional status of autophagy, which plays a critical role in senescent muscular dystrophy. As a publicly recognized strategy for extending lifespan and improving the health of the elderly, the underlying mechanisms of lifelong regular aerobic exercise for the prevention of sarcopenia have not been fully elucidated. To explore the role of lifelong aerobic exercise in the beneficial regulation of autophagic signaling pathways in senescent skeletal muscle, the natural aging mice were used as the sarcopenia model and subjected to lifelong treadmill running to evaluate corresponding parameters related to skeletal muscle atrophy and autophagic signaling pathways. Compared with the young control mice, the aged mice showed a significant reduction in skeletal muscle mass, gastrocnemius muscle weight/body weight (GMW/BW) ratio, and cross-sectional areas (CSA) of skeletal muscle fibers (*p* < 0.01). In contrast, lifelong aerobic exercise effectively rescued these reduced biomarkers associated with muscle atrophy. Moreover, as shown in the activated AMPK/PGC-1α signaling pathway, lifelong aerobic exercise successfully prevented the aging-induced impairment of the ubiquitin-proteasome system (UPS), excessive apoptosis, defective autophagy, and mitochondrial dysfunction. The exercise-induced autophagy suppressed the key regulatory components of the UPS, inhibited excessive apoptosis, and optimized mitochondrial quality control, thereby preventing and delaying aging-induced skeletal muscle atrophy.

## 1. Introduction

During the aging process, skeletal muscle shows a gradual decline in mass, strength, and movement capability, which is called sarcopenia [1]. Due to its adverse effect on the quality of life of the elderly, multiple studies have been conducted to explore effective strategies and uncover the underlying mechanistic targets for the prevention and treatments of sarcopenia. In 2016, sarcopenia was included in the World Health Organization International Classification of Diseases and officially became a new degenerative disease after osteoporosis [2]. According to the current statistical analysis, the incidence of sarcopenia in the aging population over 65 years old is 5 to 10%, and its direct pathogenic factors include various chronic diseases, physical inactivity, decreased energy intake, and reduced blood flow distribution, mitochondrial dysfunction, and increased pro-inflammatory factors in skeletal muscle [3]. As all of the above pathogenic factors are involved in the process of human aging, an urgent task for elucidating precise targets of sarcopenia during the aging process and providing an optimal interventional strategy for the prevention and treatments of sarcopenia is highly desired to realize active and healthy aging. At present, the underlying molecular mechanism for the pathogenesis of senescent skeletal muscle atrophy remains elusive. More specifically, a complete and feasible system for the prevention and treatment of aging-induced skeletal muscle atrophy has not yet been discovered.

There are limited studies on molecular mechanisms of sarcopenia that have mainly focused on inflammation, oxidative stress, apoptosis, and imbalanced protein metabolism. Recent studies have confirmed that autophagy-dependent signaling in aging-induced skeletal muscle atrophy presents obvious defects. Besides, the appropriate functional status of autophagy plays a vital role in the regulation of skeletal muscle mass and maintaining cellular homeostasis under degrading conditions [4,5]. Further studies have shown that various abnormal functions of mitochondria during the aging process of skeletal muscle are involved, such as impaired mitochondrial biogenesis, imbalanced mitochondrial dynamics, and defective mitophagy, and excessive oxidative stress [6,7]. At the same time, several studies have demonstrated that mitochondrial dysfunction can accelerate protein degradation, thus leading to an accelerated progression of skeletal muscle atrophy [8]. As a key regulator of mitochondrial biogenesis, AMPK can slow down the aging process by regulating diverse signaling pathways, and has been identified as a therapeutic target for aging-induced skeletal muscle atrophy [9]. Moreover, PGC-1α, as a downstream effector of AMPK signaling, and has been confirmed to have the function of delaying sarcopenia [10].

A growing body of studies has documented that a sedentary lifestyle is a novel risk factor for healthy living [11,12]. Most of the waking time of the elderly is spent in sedentary activities, which can result in reduced mitochondrial biogenesis, excessive apoptosis, and deficient mitochondrial turnover, thus accelerating the progression of senescent skeletal muscle atrophy [13]. In contrast, regular exercise or exercise training is the most effective mean for mitigating mass loss and strength decline of skeletal muscle in the aging population. There is irrefutable evidence that the main role of resistance exercise is to improve the quality and strength of skeletal muscle, while aerobic exercise plays a crucial role in maintaining the maximum aerobic capacity and cellular homeostasis of the aging skeletal muscle [14]. Importantly, both exercise methods can activate autophagic signaling in the aged skeletal muscle [15]. Although systematic physical exercise has been confirmed to slow down the progression of aging-related myopathies based on the regulation of autophagy [16], the underlying molecular mechanisms remain controversial.

Aerobic exercise, as a repeated exercise involving a large number of large muscle groups, can increase energy production by mitochondria and capillary density of skeletal muscle, thus promoting oxygen consumption and cellular homeostasis, and delaying skeletal muscle atrophy [17]. Moreover, due to its convenience, operability, and diverse forms, aerobic exercise is highly beneficial for delaying the aging progression of the aging population in an active and healthy aging mode. It has been foreseen to become an important and feasible strategy for the long-term prevention and mitigation of aging-induced skeletal muscle atrophy [18]. In the present study, we speculated that lifelong aerobic exercise could induce autophagy and suppress excessive apoptosis, reduce protein degradation, and improve mitochondrial quality control through the AMPK/PGC-1α signaling pathways, thereby preventing and delaying sarcopenia.

## 2. Results

### 2.1. Lifelong Aerobic Exercise Alleviated the Reduction of Gastrocnemius Muscle Weight-Body Weight (GMW/BW) Ratio in Aged Mice

Previous analysis for the internal structure of skeletal muscle has demonstrated that aging-induced skeletal muscle atrophy is mainly manifested as the atrophy of type II fibers [19]. Therefore, we chose gastrocnemius muscle as the major experimental object for further exploration. We used GMW/BW ratio as the sarcopenia index (SI) for evaluating the atrophic degree of skeletal muscle during the aging process. Compared with the youth control (YC) group, the mice from the naturally aged (OC) group demonstrated a significantly decreased GMW/BW ratio and obvious atrophic symptoms of the skeletal muscle, indicating senescence-induced reduction of skeletal muscle mass in mice *(p* < 0.001). In contrast, the GMW/BW ratio of the mice from the lifelong aerobic exercise (OE) group showed considerable rescuing upon lifelong aerobic exercise intervention when compared with that from the OC group (*p* < 0.01). This result suggested that lifelong aerobic exercise effectively improved the SI value of the aged mice and prevented the mass decline of skeletal muscle caused by aging (Table 1).

### 2.2. Lifelong Aerobic Exercise Ameliorated the Atrophy of Skeletal Muscle Fibers in Aged Mice

**Figure 1:**
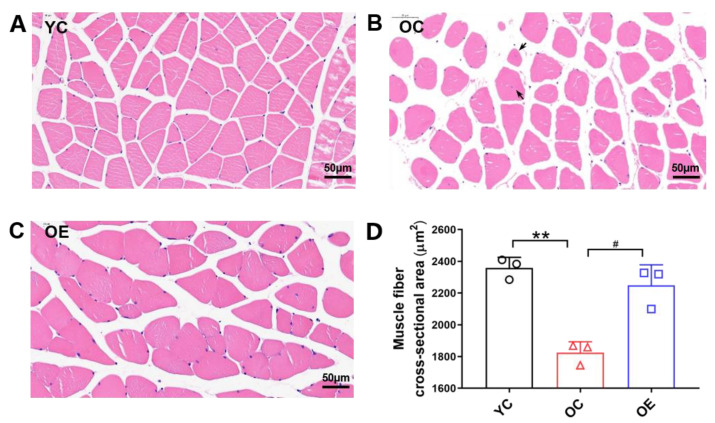
The skeletal muscle atrophy in aged mice evaluated by CSA of skeletal muscle fibers. (**A**–**C**) Representative images of skeletal muscle with HE staining (scale bar, 50 μm) and (**D**) histogram of the CSA of skeletal muscle fibers. The black arrows present nucleus-migrated fibers. More than 150 fibers were randomly selected from at least 3 skeletal muscle samples from 3 mice in each group to measure the average CSA of skeletal fibers. All data are presented as mean ± standard deviation (M ± SD) (*n* = 3 mice per group). Group symbols: YC, black circle; OC, red triangle; OE, blue square. ** *p* < 0.01 vs. YC group; ^#^ *p* < 0.05 vs. OC group.

To better observe the morphological and structural changes of skeletal muscle fibers, hematoxylin-eosin (HE) staining and corresponding statistical analysis of gastrocnemius muscle samples were conducted. Compared with the YC group, the skeletal muscle of the mice from the OC group showed more atrophic fibers as manifested by the decreased cross-sectional areas (CSA) of skeletal muscle fibers (*p* < 0.01) and the increased number of skeletal fibers with irregular shapes, as well as the frequent nucleus aggregation (Figure 1A), suggesting that aging-induced degeneration of the skeletal muscle. In contrast, the obviously decreased number of atrophic fibers and migrated nuclei, and the enlarged CSA of skeletal muscle fibers (*p* < 0.05, Figure 1B) were observed in the mice subjected to lifelong aerobic exercise. Thus, lifelong aerobic exercise effectively rescued the declined CSA and repaired the damaged textural structure of skeletal muscle fibers in aged mice.

### 2.3. Lifelong Aerobic Exercise Suppressed Ultastructural Damage of Skeletal Muscle Fibers in Aged Mice

**Figure 2:**
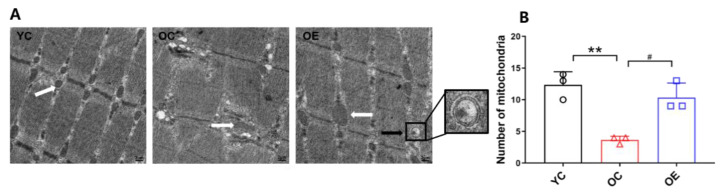
Lifelong aerobic exercise repaired the damaged mitochondria in skeletal muscle fibers during the aging process. (**A**) Representative images of gastrocnemius muscles of the mice from each group (8000×) observed by TEM; white arrows: mitochondria; black arrows: autophagosomes. (**B**) The number of mitochondria was counted from randomly selected images of 3 skeletal muscle samples from each group. All data are presented as mean ± standard deviation (M ± SD) (*n* = 3 mice per group). Group symbols: YC, black circle; OC, red triangle; OE, blue square. ** *p* < 0.01 vs. YC group; ^#^ *p* < 0.05 vs. OC group.

The typical morphological characteristics of aged skeletal muscle include a high population of fragmented or swollen mitochondria, disordered arrangement of myofilaments, or damaged fiber structure [20]. The evaluation based on transmission electron microscope (TEM) revealed a disordered arrangement of myofilaments, a large number of vacuoles with different sizes, and the swollen and abnormally shaped mitochondria in the skeletal muscle of the mice from the OC group when compared with those in the mice from the YC group (Figure 2A). Besides, a significantly lower number of mitochondria in the skeletal muscle were observed in the mice from the OC group when compared with the YC group (*p* < 0.01, Figure 2B). The skeletal muscle of the mice from the OE group had an ordered arrangement of myofilaments, a large number of normal mitochondria (*p* < 0.05), and an accumulation of autophagosomes. These findings portrayed that lifelong aerobic exercise increased the number of mitochondria and improved their structure, which could be partially due to the induction of autophagy.

### 2.4. Lifelong Aerobic Exercise Improved Mitochondrial Function and Attenuated Oxidative Stress in Aged Skeletal Muscle

**Figure 3:**
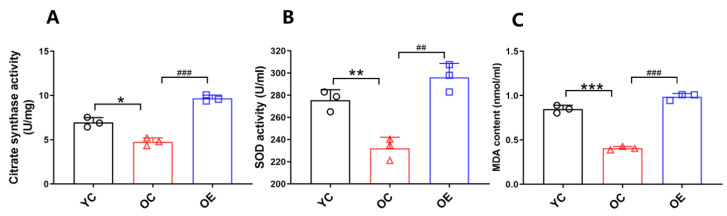
Lifelong aerobic exercise stimulated the increase of CS activity (**A**) in the skeletal muscle, and the increase of SOD activity (**B**) in serum, as well as the reduction of MDA level (**C**) in skeletal muscle. All data are presented as mean ± standard deviation (M ± SD) (*n* = 3 mice per group). Group symbols: YC, black circle; OC, red triangle; OE, blue square. *** *p* < 0.001, ** *p* < 0.01 and * *p* < 0.05 vs. YC group; ^###^ *p* < 0.001, ^##^ *p* < 0.01 vs. OC group.

In order to obtain an index of mitochondrial content, we measured the activity of mitochondrial enzyme citrate synthase (CS), one of the biomarkers of oxidative energy metabolism. Compared with the YC group, the CS activity in skeletal muscle of the mice from the OC group was significantly reduced (*p* < 0.05); in contrast, lifelong aerobic exercise stimulated the increase of CS activity (*p* < 0.001, Figure 3A). Correspondingly, in order to evaluate aging-induced oxidative stress, the activity of superoxide dismutase (SOD) and the content of malondialdehyde (MDA) in serum and skeletal muscle of the mice from each group were determined. SOD activity in serum of the mice from the OC group was significantly decreased relative to that of the mice in the YC group (*p* < 0.01, Figure 3B), which was successfully rescued upon lifelong aerobic exercise intervention (*p* < 0.01). When MDA content in gastrocnemius muscle was compared between OC and YC groups, a marked increase was observed in the OC group (*p* < 0.001). However, the MDA level in the gastrocnemius muscle of the mice from the OE group exhibited a significant reduction when compared with the OC group (*p* < 0.001, Figure 3C). From these data, it could be concluded that lifelong aerobic exercise could offset aging-induced functional reduction of mitochondria, which may be due to the improvement of mitochondrial content, and corresponding inhibition of oxidative stress.

### 2.5. Lifelong Aerobic Exercise Suppressed E3 Ubiquitin Ligases and Decreased Akt/mTOR Signaling Pathway in Aged Skeletal Muscle

**Figure 4:**
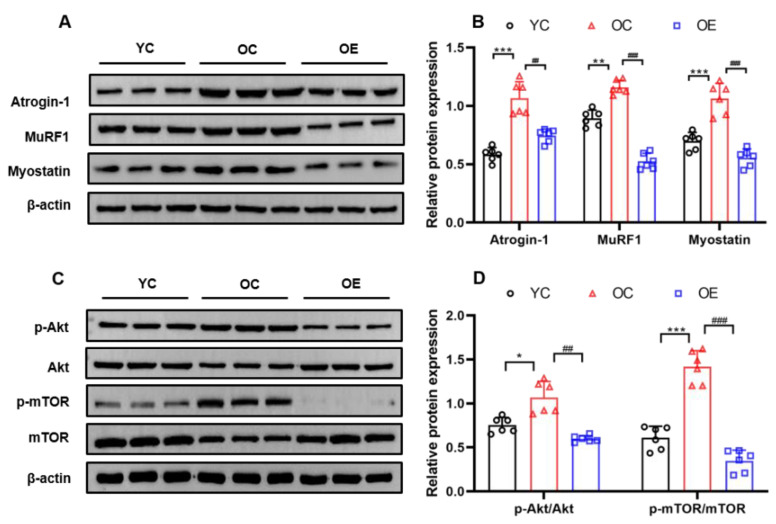
Western blot images suggested the down-regulation of Atrogin-1, MuRF1, and myostatin (**A**,**B**), and p-Akt^Ser473^/Akt and p-mTOR^Ser2448^/mTOR (**C**,**D**) associated with protein synthesis in gastrocnemius muscle of the aged mice upon lifelong aerobic exercise intervention. Equal protein loading was confirmed by β-actin. All data are presented as mean ± standard deviation (M ± SD) (*n* = 6 mice per group). Group symbols: YC, black circle; OC, red triangle; OE, blue square. *** *p* < 0.001, ** *p* < 0.01 and * *p* < 0.05 vs. YC group; ^###^ *p* < 0.001, ^##^ *p* < 0.01 vs. OC group.

Skeletal muscle homeostasis is maintained by a dynamic equilibrium between protein degradation and protein synthesis. Previous studies have actually demonstrated that E3 ubiquitin ligases (Atrogin-1/MAFbx and MuRF1) play an essential role in regulating protein ubiquitination during the atrophic process of aged skeletal muscle [21]. Myostatin is essential for the regulation of skeletal muscle mass and a negative regulator of skeletal muscle growth. Our findings suggested that Atrogin-1/MAFbx (*p* < 0.001), MuRF1 (*p* < 0.01) and myostatin (*p* < 0.001) associated with the ubiquitin-proteasome system (UPS) and ubiquitinated protein in the sarcopenic muscle of the mice from the OC group were extraordinarily up-regulated when compared with the YC group. However, lifelong aerobic exercise significantly suppressed the expression of Atrogin-1/MAFbx (*p* < 0.01), MuRF1 (*p* < 0.001) and myostatin (*p* < 0.001, Figure 4A,B), suggesting that lifelong aerobic exercise can effectively down-regulate the key regulatory components of the UPS signaling pathway, thereby alleviating protein degradation of aged skeletal muscle.

In contrast, protein anabolic factors for promoting protein synthesis via the Akt/mTOR signaling pathway in aging process of skeletal muscle could be up-regulated, as shown in the increased p-Akt^Ser473^/Akt (*p* < 0.05) and p-mTOR^Ser2448^/mTOR (*p* < 0.001); whereas lifelong aerobic exercise suppressed their increase to indirectly maintain the mass of skeletal muscle (*p* < 0.01, *p* < 0.001, Figure 4C,D).

### 2.6. Lifelong Aerobic Exercise Activated Autophagy and Inhibited Apoptosis in Aged Skeletal Muscle

**Figure 5:**
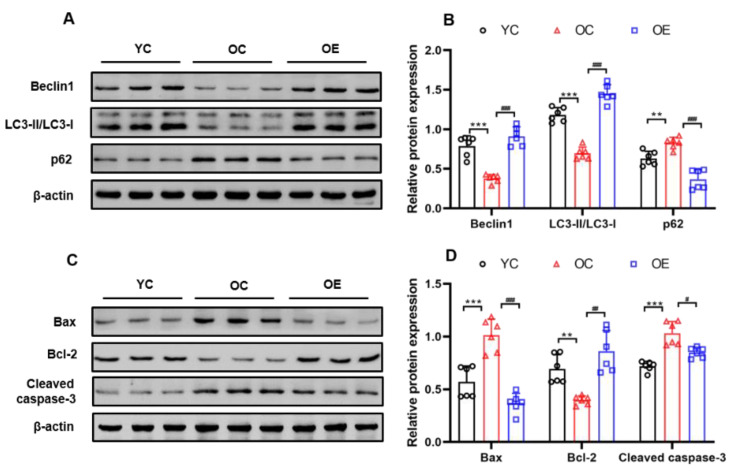
Lifelong aerobic exercise up-regulated the expression of autophagy-related protein Beclin1 and LC3-II/LC3-I ratio and down-regulated the expression of p62 (**A**,**B**); as well as down-regulated the expression of apoptosis-related proteins such as Bax and cleaved caspase-3 and up-regulated the expression of Bcl-2 (**C**,**D**) in gastrocnemius muscle of the aged mice evaluated by Western blot. Equal protein loading was confirmed by β-actin. All data are presented as mean ± standard deviation (M ± SD) (*n* = 6 mice per group). Group symbols: YC, black circle; OC, red triangle; OE, blue square. *** *p* < 0.001, ** *p* < 0.01 vs. YC group; ^###^ *p* < 0.001, ^##^ *p* < 0.01 and ^#^ *p* < 0.05 vs. OC group.

During the natural aging process, deficient autophagy in skeletal muscle is often observed, while apoptosis usually exhibits a higher state [16]. Beclin1 is a crucial protein that regulates the initiation and nucleation of autophagy; LC3-I is esterified to LC3-II under the action of critical genes for the formation of autophagic vesicles. LC3-II level represents the formation or accumulation of autophagosomes in skeletal muscle. Hence, LC3-II/LC3-I ratio can be used as a golden biomarker for the formation of autophagosomes [22]. p62, as cargo receptor between LC3 protein and ubiquitinated substrates, is selectively degraded in autolysosomes, thereby facilitating autophagic clearance and cellular recycling [23]. To better support our hypothesis, we determined the LC3-II/LC3-I ratio and the expression levels of p62 and other autophagy-related proteins to assess the activation of autophagy (Figure 5A,B). Compared with the YC group, the expression of Beclin1 and LC3-II/LC3-I ratio in the sarcopenic muscle of the mice from the OC group was significantly decreased (*p* < 0.001), and the expression level of p62 was markedly increased (*p* < 0.01). In contrast, the expression of Beclin1 (*p* < 0.001) and the LC3-II/LC3-I ratio (*p* < 0.001) associated with autophagy in the skeletal muscle of the mice from the OE group was significantly up-regulated, and the expression of p62 was significantly down-regulated (*p* < 0.001), indicating that lifelong aerobic exercise successfully activated autophagy or rescued the abnormal functional status of autophagy in skeletal muscle of the aged mice.

In addition, autophagy and apoptosis are highly regulated as the mutually inhibited and interrelated biological processes. Excessive apoptosis can lead to the declined function of autophagy and cellular degradation, thereby inducing skeletal muscle atrophy and corresponding myopathies. In the present study, as the central modulators of intrinsic apoptosis, the expression of pro-apoptotic biomarkers such as Bax (*p* < 0.001) and cleaved caspase-3 (*p* < 0.001) in the sarcopenic muscle of the mice from the OC group was significantly increased, and the expression of an anti-apoptotic biomarker such as Bcl-2 was significantly decreased (*p* < 0.05). Nevertheless, lifelong aerobic exercise notably attenuated apoptotic cell death, as shown by a significant decrease in the expression of Bax (*p* < 0.001) and cleaved caspase-3 (*p* < 0.05), and an increase in the expression of Bcl-2 (*p* < 0.01) in the skeletal muscle of the mice from the OE group. Therefore, lifelong aerobic exercise could adequately suppress apoptotic signaling and improve anti-apoptotic capacity of the aging skeletal muscle (Figure 5C,D).

### 2.7. Lifelong Aerobic Exercise Improved Mitochondrial Quality Control via Activating the AMPK/PGC-1α Signaling Pathway in Aged Skeletal Muscle

**Figure 6:**
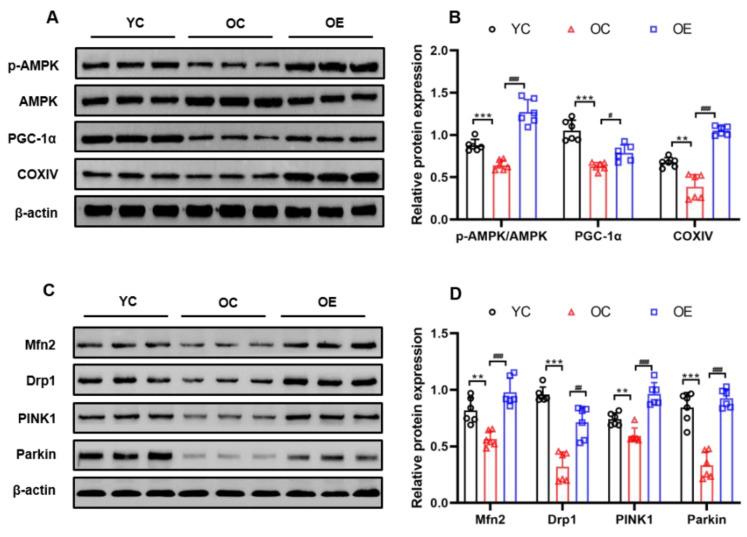
Lifelong aerobic exercise up-regulated the expression of the proteins such as p-AMPK ^Thr172^/AMPK, PGC-1α and COXIV associated with mitochondrial biogenesis (**A**,**B**) and the proteins including Mfn2, Drp1, PINK1, and Parkin (**C,D**) associated with mitochondrial quality control in gastrocnemius muscle of the aged mice evaluated by Western blot. Equal protein loading was confirmed by β-actin. All data are presented as mean ± standard deviation (M ± SD) (*n* = 6 mice per group). Group symbols: YC, black circle; OC, red triangle; OE, blue square. *** *p* < 0.001, ** *p* < 0.01 vs. YC group; ^###^ *p* < 0.001, ^##^ *p* < 0.01 and ^#^ *p* < 0.05 vs. OC group.

In order to elucidate the potential underlying mechanisms of lifelong aerobic exercise for the prevention and treatment of muscular atrophy, the signaling pathways involved in AMPK/PGC-1α-mediated mitochondrial quality control were explored. AMPK is a vital metabolic regulator that modulates muscle plasticity and mitochondrial function in skeletal muscle [24]. PGC-1α plays a pivotal role in the regulation of mitochondrial biogenesis since it connects the external stimulation signaling to regulate mitochondrial function [10]. Our results identified the significantly reduced expression of PGC-1α (*p* < 0.001) and p-AMPK^Thr172^/AMPK ratio (*p* < 0.001), as well as mitochondrial respiratory chain key protease cytochrome C oxidase subunit IV (COXIV) (*p* < 0. 01) associated with mitochondrial biogenesis in the sarcopenic muscle of the aged mice (Figure 6A,B). Besides, the mitophagy-related proteins such as PINK1 (*p* < 0.01) and Parkin (*p* < 0.001) were also significantly decreased in the skeletal muscle of the aged mice (Figure 6C,D). Similarly, a dramatic reduction in the expression of mitochondrial dynamics-related proteins such as mitofusion 2 (Mfn2) (*p* < 0.01) and dynamin-related protein 1 (Drp1) (*p* < 0.001) were observed in the sarcopenic muscle of the aged mice. In contrast, lifelong aerobic exercise up-regulated the expression of these proteins associated with mitochondrial biogenesis, mitophagy, and mitochondrial dynamics in the sarcopenic muscle of the aged mice, suggesting that lifelong aerobic exercise can enhance mitochondrial quality control via the AMPK/PGC-1α signaling pathway.

## 3. Discussion

In the present study, we have implemented lifelong aerobic exercise in laboratory ICR mice, which have a much shorter life cycle than humans. The 50% survival, mean survival, and maximal survival ages of ICR mice were 20, 18, and 31 months, respectively [25]. Thus, we adopted a 17-month-old natural aging mouse model and found that the SI value of the mice in the natural aging model group was significantly lower than that in the young control group, indicating that the aged mice can be regarded as a model of sarcopenia [26]. Meanwhile, the CSA of skeletal muscle, as the most intuitive indicator of sarcopenia, combined with the increased mitochondrial content, further verified the above results, indicating that lifelong aerobic exercise can effectively delay the atrophy of skeletal muscle in the aging process of mice.

Increasing evidence has demonstrated that the imbalance of protein synthesis and protein degradation during the aging process can lead to the declined mass of skeletal muscle, a common occurrence in senile muscular atrophy [27]. Protein degradation controlled by the UPS plays a dominant role in aged skeletal muscle [28]. Two E3 ubiquitin ligases, including Atrogin-1 and MuRF1, the most up-regulated proteins under the conditions of skeletal muscle atrophy, are the sensitive biomarkers for protein degradation by the UPS [21]. In the present study, one feature in the aged mice was the increase in Atrogin-1, MuRF1, and myostatin triggered by aging, indicating the over-activation of UPS. However, followed by lifelong aerobic exercise, the key regulatory components of the UPS signaling pathway in sarcopenic muscle were reduced, and the aging-associated activation of catabolic signaling was suppressed, thereby postponing muscular atrophy. On the other hand, it is well accepted that the activation of the Akt/mTOR signaling pathway facilitates the growth of skeletal muscle [29]. To our surprise, our results suggested that aging could increase the expression of p-Akt^Ser473^ and p-mTOR^Ser2448^, indicating that common anabolic resistance might occur in senile muscular atrophy [30]. However, lifelong aerobic exercise-induced restoration of Akt^Ser473^ and mTOR^Ser2448^ hyper-phosphorylation improves insulin signaling in skeletal muscle of older animals and indirectly maintains their skeletal muscle mass [31]. Further studies are needed to explore a definite link between Akt and mTOR phosphorylation, insulin signaling, anabolic resistance, and muscle mass in skeletal muscle of older animals. Nevertheless, in this study, aged skeletal muscle suggested marked protein ubiquitination, along with decreased mass and cross-section area of skeletal muscle fibers, confirming that decreased protein degradation, rather than increased protein synthesis, is the main effect of lifelong aerobic exercise on the prevention and treatment of senile muscular atrophy.

Some recent reports have verified autophagy-dependent signaling transduction defects in skeletal muscle at the aging state, and the appropriate functional status of autophagy plays a crucial role in controlling skeletal muscle mass under degradation conditions [32,33]. Besides, skeletal muscle is the most vigorous metabolic organ in the human body. Exercise-induced autophagy in the skeletal muscle can accelerate the elimination and recycling of damaged proteins or misfolded organelles, improve metabolism, and stabilize the cellular homeostasis of skeletal muscle [34]. Moreover, aerobic exercise can suppress skeletal muscle atrophy by improving the functional status of autophagy, as confirmed by the expression levels of multiple autophagy-related biomarkers, including Atg5, Atg7, p62, and LC3-II in the aging rat model [35]. Another study has also documented that after 8 weeks of treadmill running, the down-regulation of Beclin1 and Atg7 in aged rats caused by aging was offset, and the quality of skeletal muscle was also improved effectively [36]. Consistent with these findings, we observed an increased Beclin1 and LC3-II/LC3-I ratio and reduced p62 in the mice upon lifelong aerobic exercise intervention. This finding suggested that aerobic exercise could enhance autophagic flux, accelerate the removal of intracellular damaged proteins and metabolic wastes in cells, thereby promoting the structural and functional remodeling of skeletal muscle fibers.

As the age increases, there is a continuous decrease in quantity and quality of mitochondria, excessive oxidative stress, and imbalanced activity between pro-apoptotic protein Bax and anti-apoptotic protein Bcl-2 in skeletal muscle. This leads to increased permeability of the outer mitochondrial membrane and an enhanced release of cytochrome C, which in turn activates caspase-3 and promotes protein degradation and apoptosis in the skeletal muscle [37]. Similarly, aging activates the mitochondria-dependent apoptotic pathways in the aged rats as a result of down-regulated Bcl-2 and up-regulated Bax along with increased cleaved caspase-3 [38]. Meanwhile, apoptosis-mediated degradation is thought to be associated with the aging process of skeletal muscle [39], and increased apoptosis can activate E3 ligases Atrogin-1 and MuRF1, thus causing increased protein degradation and exacerbated aging-induced skeletal muscle loss [40]. On the other hand, caloric restriction, exercise intervention, drug administration, and gene therapy can hinder the excessive apoptosis of skeletal muscle cells, maintain skeletal muscle mass and integrity, and improve its physical performance [41]. Correspondingly, our results clearly indicated that long-term regular aerobic exercise can effectively mitigate the degree of apoptosis due to the up-regulated Bcl-2 and down-regulated Bax along with decreased cleaved caspase-3 in the skeletal muscle of the aged mice.

An important feature of aging is low energy metabolism, and the skeletal muscle is the primary organ system in the energy metabolism of human body. Dysfunctional metabolism in the skeletal muscle can accelerate its aging progression. AMPK/PGC-1α signaling pathway plays a critical role in regulating energy metabolism and function in skeletal muscle [42]. AMPK, as a conserved serine/threonine protein kinase and a crucial regulator of metabolic energy sensors, can maintain the stability of the energy state by sensing the changes in intracellular AMP/ATP ratio, thus changing the expression of its downstream proteins and regulates the energy production and consumption in the body [9]. Along with the increase in age, AMPK changes are associated with many metabolic diseases. The activated AMPK can regulate the energy metabolism-related signaling pathways to promote mitochondrial biogenesis and improve the function of the aging skeletal muscle with disorders [26]. AMPK is a key regulator of mitochondrial function and oxidative stress in skeletal muscle by regulating the mitochondrial quality control system [43]. PGC-1α is a junction between external stimulus signals and internal mitochondrial functions, known as the key factor in the regulation of mitochondrial biogenesis [10]. Recently, AMPK has been identified to stimulate mitochondrial biogenesis through up-regulated PGC-1α [44]. Exercise can lead to the targeting of mitochondria for mitophagy and significantly increased autophagy and mitophagy flux. This effect is significantly attenuated in PGC-1α-deficient mice [18]. Additionally, the overexpression of PGC-1α has been reported to prevent mitochondria-mediated apoptosis [45], and PGC-1α knockout mice show muscular atrophy [46]. Similar to previous studies, our results further confirmed that mitochondrial dysfunction and the AMPK/PGC-1α signaling pathway is significantly inhibited in skeletal muscle at the aging state. Moreover, lifelong regular aerobic exercise can effectively promote mitochondrial biogenesis via the activated AMPK/PGC-1α signaling pathway and optimized balance between autophagy and apoptosis, thereby delaying aging-induced atrophy of skeletal muscle.

At the same time, the gradual decrease in physical activity and the increase in oxidative stress can cause abnormal accumulation of damaged mitochondria, and even the initiation of mitochondria-mediated apoptosis. This ultimately causes the dysfunctional loss of skeletal muscle in the elderly. Similarly, elevated MDA level, along with decreased SOD and CS activity in aging skeletal muscle, is a clear indicator of oxidative stress. To cope with these changes, mitophagy, as a kind of selective autophagy, can identify and degrade damaged and dysfunctional mitochondria, thus generating a self-protective coping mechanism against mitochondrial dysfunction [47]. PINK1/Parkin is one of the components in the classic signaling pathway of mitophagy. Under the conditions of stress, injury or aging, mitochondrial kinase PINK1 can gather outside the membrane of dysfunctional mitochondria and translocate them into it selectively by phosphorylating and recruiting Parkin, and then ubiquitinate outer mitochondrial membrane proteins such as mitochondrial fusion key proteins Mfn1/2 and fission key protein Drp1 to participate in mitophagy [48]. In this study, long-term aerobic exercise offset the mass and strength decline caused by aging through enhancing mitochondrial self-regulation and repairing capability and reducing oxidative stress, thereby postponing the aging-induced atrophy of skeletal muscle.

On the other hand, mitochondrial dysfunction, especially metabolic activity, contributes many disorders, including metabolic diseases and aging process. Exercise can remarkably improve the quantity and quality of mitochondria through several adaptive processes, such as mitochondrial dynamics and mitophagy [49]. Our findings are in agreement with several previous studies that mitochondrial dynamics not only regulates mitochondrial number and morphology, but also plays a crucial role in preventing mitochondrial fragmentation and excessive oxidative stress, thus responding to apoptotic stimuli and mitochondrial quality control [50]. Mfn1/2 and Drp1 are cooperated to maintain the dynamic changes of mitochondrial junction structure. Previous studies have found that Mfn2 deficiency in skeletal muscle exacerbates aging-induced mitochondrial dysfunction in the skeletal muscle associated with the accumulation of mitochondrial damage, indicating Mfn2 deficiency as the important trigger of mitochondrial dysfunction during the aging process [50]. Similarly, recent publications have reported the volume-enlarged or swollen mitochondria in Drp1 knockout skeletal muscle tissue, indicating that the absence of Drp1 can seriously affect mitochondrial morphology and function [51]. In the current study, we found that long-term aerobic exercise offset the decreased expression of mitochondrial fusion and fission biomarkers such as Mfn2 and Drp1 during the aging process. Thus, long-term aerobic exercise increases steady state or dynamic balance of mitochondrial fusion and fission to improve the movement efficiency of the mitochondrial network for adapting to the changing cellular environment.

## 4. Materials and Methods

### 4.1. Animals, Study Design, and Ethics

Three-month-old ICR male mice (body weight, 46.7 ± 2.3 g at the beginning of the experiment; Certificate No. 2000600024096) were purchased from the Experimental Animal Center for Hubei Provincial Center for Disease Control and Prevention (Wuhan, China). The mice were randomly divided into two groups: the control group (OC; *n* = 10; naturally aged model group) and the lifelong aerobic exercise group (OE; *n* = 10) with corresponding exercise interventions (subjected to lifelong treadmill running) until the age of 17 months old. Another 10 three-month-old male ICR mice were purchased and used as the youth control group (YC) after the natural aging model was successfully constructed. All mice were housed in a specific pathogen-free (SPF) environment with conditions of standard light-dark cycle (12 h–12 h) and temperature (22 ± 2 °C). All mice were supplied with a standard laboratory chow (70.77% kcal from carbohydrate, 20.7% kcal from protein crude, 4.15% kcal from fat crude, 2.31% kcal from fiber crude, 1.24% kcal from calcium, and 0.83% kcal from phosphorus) (WQJX, Wuhan, China) and water ad libitum. All procedures performed in this study involving animals were in accordance with the ethical standards of the Animal Care and Use Committee at Wuhan Sports University and approved by this committee (Code No. 2017-020).

### 4.2. Aerobic Exercise Protocol

This experimental intervention time for the mice is at a fixed time from 18:00 pm to 21:00 pm during each intervention period. All mice from the OE group were habituated to treadmill running for 1 week. During this period, the running duration and intensity were progressively increased at the increment of 4.2 m/min until the running duration of 60 min/day at the speed of 12 m/min. After the habituation period, the mice from the OE group were subjected to treadmill running at the speed of 12 m/min (moderate-intensity exercise) for 60 min/day and 5 days/week [52] for 55 consecutive weeks until 17 months old as the aged mice. After the modeling of the aged mice, the body weights of the aged mice were measured and recorded, and all mice were sacrificed by cervical dislocation. The gastrocnemius muscle tissues of the left and right hind limbs of the mice were collected and measured for the subsequent assessment of SI, respectively, and then immediately frozen in liquid nitrogen and stored at −80 °C freezer for later use.

### 4.3. Histological Examination of Gastrocnemius Muscle

Gastrocnemius muscle samples from YC, OC, and OE groups (n = 3 mice per group) were immediately harvested, fixed in 10% neutral-buffered formalin, and cut into the slices in the thickness of 5 µm using a cryotome. Tissue slices were dewaxed, rehydrated, and stained with HE to analyze the morphology and the damage of gastrocnemius muscle. Images were observed under a light microscope (BX51TF OLYMPUS, Tokyo, Japan) at 400× magnification. The total area of skeletal muscle fibers (μm^2^) of gastrocnemius muscle (3 randomly selected images per individual) was determined using Image-ProPlus 6.0 software (Media Cybernetics, Bethesda, MD, USA) with careful manual annotations and 40–70 muscle fibers (pieces) per image were counted. At least 150 skeletal muscle fibers were counted in each group for quantification, and skeletal muscle fibers in different regions were randomly selected and analyzed in a blinded mode. The average CSA of gastrocnemius muscle fibers (μm^2^) was calculated using the formula: total muscle fiber area (μm^2^)/number of muscle fibers.

### 4.4. Transmission Electron Microscopic Examination

The transmission electron microscopic assay was executed as illustrated previously [53]. The gastrocnemius muscle samples at the volume of 1 mm^3^ were fixed, rinsed, cut (50 nm), and stained. The ultrastructure of gastrocnemius muscle during the aging process with corresponding exercise intervention was then evaluated and analyzed under a transmission electron microscope (HT7700 Hitachi, Tokyo, Japan) at the Research Center for Medicine and Structural Biology of Wuhan University. To quantify the number of mitochondria in gastrocnemius muscle, 3 electron micrographs were acquired at 8000× from ultrathin sections of each sample. A suitable field of view was randomly selected. The Image-ProPlus 6.0 software (Media Cybernetics, Bethesda, MD, USA) was used to manually quantify the number of mitochondria, with at least three image areas for each sample in each group.

### 4.5. Mitochondrial Enzyme Activity

The maximal activity of CS in gastrocnemius muscle tissue was determined by measuring coenzyme A formation at 412 nm with citrate synthase activity assay kit (Solarbio BC 1060, Beijing, China) according to the manufacturer’s instructions.

### 4.6. SOD Activity and MDA Content in Serum and Gastrocnemius Muscle

The activity of SOD in serum was also assayed using a SOD assay kit (A001-3, Nanjing Jiancheng Company, Nanjing, China) according to the manufacturer’s instructions. Briefly, the reaction was initiated by the addition of pyrogallol, and the absorbance was measured kinetically at 450 nm, 37 °C, for 20 min. The activity of SOD was expressed as U/mg protein, where one unit of the enzyme was defined as the amount of the sample for inhibiting the pyrogallol oxidation by 50%. Similarly, the content of MDA, in gastrocnemius tissue, was detected by using a lipid peroxidation (malondialdehyde; MDA) assay kit (A003-1, Nanjing Jiancheng Company, Nanjing, China). Lipid peroxidation was determined by thiobarbituric acid (TBA) reactive substance tests. In brief, each sample was homogenized in a certain volume (10% *w/v*) of ice-chilled 50 mM phosphate buffer (pH 7.4). After centrifugation at 3000× *g* at 4 °C for 10 min, the supernatants were collected. The absorbance of each supernatant sample was measured at 532 nm. The results were expressed as nmol MDA in each gram of skeletal muscle samples.

### 4.7. Western Blotting

Western blotting was performed as described previously [54]. The gastrocnemius muscle samples were sequentially subjected to washing with PBS buffer twice, RIPA lysis, and homogenization on ice in the presence of protease inhibitor, phenylmethylsulfonyl fluoride (PMSF). The homogenate was subjected to centrifugation for 10 min at 15,000× *g* at 4 °C. Aliquots of the supernatant were frozen at −80 °C, with a portion of these homogenates used by BCA kit (Walterson Biotechnology Inc., Beijing, China) for the determination of the total protein. The prepared protein samples were boiled in a water bath at 95 °C for 5 min. The soluble protein was separated using 10% or 12% sodium dodecyl sulfate-polyacrylamide gel (SDS-PAGE) and then transferred to a polyvinylidene fluoride (PVDF) membrane. The target protein in the membrane was probed by specific primary antibody against cleaved caspase-3 (ab2302), PGC-1α (ab54481), myostatin (ab124721), COXIV (ab14744), Atrogin-1 (ab168372), and MuRF1 (ab183094; 1:1000; Abcam, Cambridge, MA, USA), Beclin1 (#3495; 1:1000), p62 (SQSTM1/Sequestome 1; #23214; 1:1000), LC3 (microtubule-associated protein-1 light chain 3, LC3; #4108; 1:1000), Bcl-2 (#3498; 1:1000), Bax (#14796; 1:1000), AMPK (#5831; 1:1000), p-AMPK^Thr172^ (#2535; 1:1000), Akt (#4685; 1:1000), p-Akt^Ser473^ (#4060; 1:1000), mTOR (#2983; 1:1000), p-mTOR^Ser2448^ (#5536; 1:1000), Drp1 (#8570; 1:1000), Mfn2 (#9482; 1:1000), PINK1 (#6946; 1:1000), Parkin (#2132; 1:1000), and β-actin (#4970; 1:1000; Cell Signaling Technology, Beverly, MA, USA); as well as the corresponding secondary antibody (BA1054 and BA1051; 1:5000; Boster Bio, Wuhan, China). Protein bands were visualized using enhanced chemiluminescence (ECL) reagent and imaged using an ultra-sensitive fluorescence/chemiluminescence imaging system ChemiScope6300 (CLiNX Science Instruments, Shanghai, China).

### 4.8. Statistical Analysis

All data were expressed as mean ± standard deviation (M ± SD). Statistical analysis was performed using GraphPad Prism 9.0.0 software (La Jolla, CA, USA). Then, for parametric data, one-way analysis of variance (ANOVA) was used to analyze the statistical significances of differences between multiple groups. Otherwise, non-parametric Kruskal-Wallis analysis was performed. For all tests the significance level was set at *p* < 0.05. The analysis of images was conducted by a single investigator blinded to the samples.

## 5. Conclusions

**Figure 7:**
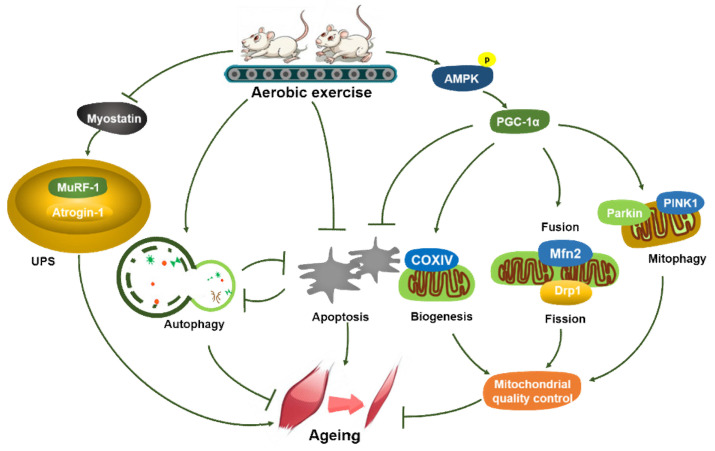
Lifelong aerobic exercise alleviates the initiation and progression of sarcopenia by activating autophagy/mitophagy and inhibiting excessive apoptosis to improve mitochondrial quality control and suppress UPS-mediated protein degradation via the AMPK/PGC-1α signaling pathway.

Lifelong aerobic exercise can suppress the decline in the mass of skeletal muscle of aged mice and improve the ultrastructure of aged myofibrils through stimulating mitochondrial biogenesis, optimizing mitochondrial dynamics, and rescuing abnormal functional status of autophagy/mitophagy via the AMPK/PGC-1α signaling pathway, to repair dysfunctional mitochondria, improve energy metabolism, rescue deficient autophagy, suppress excessive apoptosis, enhance mitochondrial quality control, and inhibit protein ubiquitination, thereby preventing or delaying aging-induced atrophy of skeletal muscle (Figure 7).

## Figures and Tables

**Table 1:** Lifelong aerobic exercise alleviated the reduction of the GMW/BW ratio of aged mice.

Groups	Body Weight (g)	Gastrocnemius Muscle (g)	GMW/BW Ratio (×100)
YC	46.733 ± 2.375	0.433 ± 0.0296	0.9261 ± 0.064
OC	57.851 ± 4.324	0.451 ± 0.0384	0.7801 ± 0.050 ***
OE	51.709 ± 3.284	0.434 ± 0.0377	0.8395 ± 0.051 ^##^

Note: The skeletal muscle atrophy of the aged mice was evaluated based on GMW/BW ratio. All data are presented as mean ± standard deviation (M ± SD) (*n* = 10 mice per group). *** *p* < 0.001 vs. YC group; ^##^ *p* < 0.01 vs. OC group.

## Data Availability

The data presented in this study are available in article.
